# Intestinal parasites: Associations with intestinal and systemic inflammation

**DOI:** 10.1111/pim.12518

**Published:** 2018-03-04

**Authors:** G. A. Zavala, O. P. García, M. Camacho, D. Ronquillo, M. Campos‐Ponce, C. Doak, K. Polman, J. L. Rosado

**Affiliations:** ^1^ Facultad de Ciencias Naturales Universidad Autónoma de Querétaro Querétaro Mexico; ^2^ Faculty of Earth & Life Sciences Vrije Universiteit Amsterdam Amsterdam The Netherlands; ^3^ Department of Biomedical Sciences Institute of Tropical Medicine Antwerp Antwerp Belgium

**Keywords:** cytokines, helminths, intestinal inflammation, intestinal parasites, leptin, protozoa, systemic inflammation

## Abstract

The aim of the present study was to evaluate associations between intestinal parasitic infection with intestinal and systemic inflammatory markers in school‐aged children with high rates of obesity. Plasma concentrations of C‐Reactive Protein (CRP), leptin, TNF‐α, IL‐6 and IL‐10 were measured as systemic inflammation markers and count of stool leukocytes as marker of intestinal inflammation in 291 children (6‐10 years). Intestinal parasitic infection was measured by stool examination. Logistic regression analyses were performed to determine the odds of having high inflammatory markers for each parasite or group of parasites as compared to parasite‐free children while adjusting for sex, age, mother's educational level and percentage of body fat. The prevalence of soil‐transmitted helminths and intestinal protozoa infections was 12% and 36%, respectively. Parasitic infection was not associated with CRP, IL‐6, IL‐10 or TNF‐α. Children infected with *Ascaris lumbricoides* (aOR: 5.91, 95% CI: 1.97‐17.70) and *Entamoeba coli* (aOR: 8.46, 95% CI: 2.85‐25.14) were more likely to have higher stool leucocytes than parasite‐free children. Children with multiple infections (aOR: 10.60, 95% CI: 2.85‐25.14) were more likely to have higher leptin concentrations than parasite‐free children. Intestinal parasitic infection was not associated with systemic inflammation, but was associated with intestinal inflammation. Having multiple infections were associated with higher leptin concentrations.

## 1 INTRODUCTION

Mexico is undergoing a nutritional transition, with rising rates of obesity and related chronic diseases.^1^, ^2^ Childhood obesity in particular is considered to be a challenging health problem. High body fat stimulates systemic inflammation via an increased secretion of inflammatory molecules, most importantly IL‐6 and tumour necrosis factor‐alpha (TNF‐α), which is the mechanism by which obesity relates to chronic disease.^3^ Regulatory markers such as IL‐10 dampen the obesity‐related inflammatory response.^4^ In addition to the obesity challenge, parasitic infections are also an important health problem in Mexico; it is estimated that half of the paediatric population is infected with at least one species of intestinal parasite.^5^, ^6^ Interestingly, these parasites have been associated with the same molecules involved in the systemic inflammatory process.^7^, ^8^ For instance, soil‐transmitted helminths (STHs) can regulate systemic inflammation via a response dominated by anti‐inflammatory cytokines such as IL‐10, regulating the concentration of the inflammatory cytokines secreted by adipose tissue such as TNF‐α, IL‐6 and leptin.^9^, ^10^, ^11^ In addition to its role in inflammation processes, leptin is a hormone with other roles in human metabolism such as regulation of energy intake/expenditure, hematopoiesis and gut permeability.^12^, ^13^, ^14^, ^15^

In contrast to STHs, less is known about the association between intestinal protozoa with systemic inflammation. In vitro and animal studies have shown that intestinal protozoa such as *Blastocystis* and *Entamoeba histolytica* trigger TNF‐α promoting an inflammatory response, but the mechanisms are not known.

Soil‐transmitted helminths have shown to regulate intestinal inflammation by the secretion of regulatory molecules such as IL‐10 by white blood cells.^16^ On the other hand, pathogenic protozoa have shown to cause intestinal inflammation and tissue damage,^17^, ^18^ which have shown to exacerbate the systemic inflammatory process.^19^

Given the high rates of childhood obesity and parasitic infection in Mexico and the effects both have on inflammatory reactions, intestinal parasites may be associated with systemic and intestinal inflammation.^12^, ^13^, ^14^, ^15^ The aim of this study was to evaluate the associations between intestinal parasites with intestinal and systemic inflammation in a population of Mexican school children with high prevalence of obesity.

## 2 METHODS

### 2.1 Subjects and experimental design

A total of 291 children (6‐10 years of age) participated in this cross‐sectional study from February to May of 2013. The children were randomly selected from the local school of the rural community of “Santa Cruz” in Queretaro, Mexico. The children's legal guardians received oral and written information about the study, and were asked to sign an informed consent letter. Children who had received any treatment against intestinal parasites in the last 4 months or with any physical or mental disability were excluded from the study. The study was approved by the Bioethics Committee of the Universidad Autonoma de Querétaro (UAQ).

A sample size of 284 children was calculated to find differences in terms of body fat, which was studied as the main outcome in a previous study in this population.^8^ This sample size also allows to find differences in TNF‐α concentration between infected and parasite‐free children, with an estimated prevalence of infection of 20% and an estimated standard deviation of 3 pg/mL for TNF‐α, assuming a type I error of 5% and a statistical power of 80%.^20^

Once the children were selected for inclusion, legal guardians were asked to attend the community health clinic to answer a socio‐economic and medical history questionnaire, including queries of mother's educational level, sex and age of the children.

### 2.2 Systemic inflammation markers

A fasting blood sample (7 mL) was taken from each participant in the morning (7:00‐8:30 am) and collected in vacuum tubes (Becton Dickinson, Franklin Lakes, NJ, USA). Samples were centrifuged at 250 g rpm for 15 minutes (Beckman Allegra 21R, Palo Alto, CA, USA), and plasma was separated. Concentrations of leptin and C‐Reactive Protein (CRP) in plasma were measured in duplicate using commercial ELISA kits (Human Leptin Elisa Kit, Linco Research; High‐Sensitivity C‐Reactive Protein ELISA Kit, Bioquant). The concentration of the inflammatory cytokines TNF‐α, IL‐6 and the regulatory cytokine IL‐10 was measured using high‐sensitivity commercial ELISA kits (Millipore CRP ELISA, MO, USA). All ELISA kits were analysed in a Multiskan Ascent microplate photometer (Thermo Electron Corporation, MA, USA). All the biochemical analyses were performed by trained personnel at the Human Nutrition Laboratory, UAQ.

### 2.3 Parasitology and intestinal inflammation

A stool sample was collected from each participant in the morning (7:00‐8:30 am). A coproparasitological test consisting of a wet mount with iodine staining of slides was performed to screen for the presence of protozoa parasites, as described by WHO. Samples with one or more protozoa trophozoites or cysts were considered as infected. In addition, 2 Kato‐Katz smears (2 × 41.7 mg) were performed according to standard procedures to screen, to determine the presence and to quantify the number of eggs of STHs. Infection was defined as the presence of species‐specific eggs or trophozoites or cysts detected by either of the 2 methods. Children with no protozoa trophozoites or cysts, and no STH eggs were classified as parasite‐free. All children diagnosed with intestinal parasites were referred to the local health clinic for treatment.

Intestinal inflammation was measured by the count of stool leukocytes.^21^, ^22^ The samples were examined for the presence of faecal leukocytes on direct wet smears. Each sample was stained with methylene blue, and the number of leukocytes per field was recorded.^21^, ^23^ All microscopy tests were performed by a trained technician.

### 2.4 Body composition

Children and legal guardians were transported from their local communities to the Nutrition Clinic at UAQ for anthropometry and body composition measurements. Weight and height were measured in duplicate by trained and standardized personnel with a precision of 0.1 g or 0.1 cm, respectively, following World Health Organization (WHO) procedures.^24^ Weight was measured in all participants using light clothing and barefoot using a calibrated digital scale (SECA, mod 813 Hamburg, Germany); height was measured using a stadiometer (SECA, mod 206 Hamburg, Germany). Body mass index (BMI)‐for‐age *z*‐score and height‐for‐age *z*‐score (HAZ) were calculated using the AnthroPlus software (Geneva: WHO, 2009) based on the WHO criteria of BMI‐for‐age for children aged 5‐19 years. Children were considered to be underweight if they had 2 z‐scores below, overweight if they had 1 *z*‐score above and obese if they had 2 *z*‐scores above the reference median of the BMI‐for‐age *z*‐score and were considered to be stunted if they had 2 *z*‐scores below the WHO reference median of height‐for‐age *z*‐score.^25^

Whole body composition was measured by a certified technician using Dual‐energy X‐ray absorptiometry (DXA) (Hologic Mod Explorer, 4500 C/W QDR, INC 35 Crosby Drive, Bedford, MA 01730, USA). Body fat per cent and body fat content in kg were determined directly from DXA. Elevated body fat was considered above 30% for girls and above 25% for boys.^26^

### 2.5 Data analysis

#### 2.5.1 A logistic regression

In principle, all intestinal parasites were analysed separately (ie *Ascaris lumbricoides, Entamoeba coli, Endolimax nana*), unless the prevalence was below 10%, then they were grouped. Intestinal parasites with a prevalence below 10% were analysed only as part of the group with STHs infection (*A. lumbricoides* and hookworm) or intestinal protozoa infection (*E. coli, E. histolytica/dispar, E. nana, Balantidium coli, Giardia lamblia*). Children with more than 1 species of intestinal parasite were categorized and analysed as a different group called multiple infections.

The inflammatory markers were not normally distributed and therefore they were categorized as low and high concentration (below and above the median).

A logistic regression expressed as adjusted odds ratios (aOR) was carried out to determine the association between the concentration of inflammatory markers with overweight and obesity. Then a logistic regression expressed as aOR, was used to determine the association between the concentration of inflammatory markers with each parasite or parasite group separately (STHs, *A. lumbricoides*, protozoa, *E. coli, E. nana and* multiple infection) comparing them to parasite‐free children. To decrease the occurrence of type 1 errors due to multiple comparisons, the Bonferroni‐adjusted test of significance was used.^27^ Body fat (%), sex (m/f), age (y), mother's education level (y) and malnutrition (stunted or underweight) were included in the model as confounders, as these factors are associated with both elevated inflammatory markers and intestinal parasitic infection.^28^

## 3 RESULTS

The prevalence of parasitic infection in this population was 60% (Table 1). STH monoinfections were detected in 12.1% of the population while protozoa monoinfections were present in 35.6% of the population and 12.7% had multiple infections. The most common STH monoinfection was *A. lumbricoides (A. lumbricoides)*. The most prevalent intestinal protozoa monoinfections were *E*. *coli*, followed by *E. nana*. All other studied protozoa had a prevalence below 7%. We did not find any children infected with *Trichuris trichiura* or *Blastocystis hominis*. There were no differences between infected and parasite‐free children in terms of age, sex, mother's educational level and percentage of body fat.

**Table 1:** Prevalence of parasitic infection in the studied children (n = 291)

	n	
Overall infection	176	60.5%
Soil ‐transmitted helminths	35	12.1%
* Ascaris lumbricoides* a	31	10.7%
* *Hookworm^a^	4	1.4%
Protozoa	104	35.6%
* Entamoeba coli* a	37	12.7%
* Entamoeba histolytica/dispar* a	8	2.7%
* Endolimax nana* a	33	11.3%
* Balantidium coli* a	18	6.2%
* Giardia lamblia* a	8	2.7%
Multiple infections	37	12.7%

a Infected only with the specified species (monoinfection).

Among the studied children, 54% were girls. A low prevalence of underweight (1.7%) and stunting (5.5%) and a high prevalence of overweight (18.6%), obesity (9.6%) and elevated body fat (53.3%) were found. Table 2 summarizes the general characteristics of the children who participated in the study.

**Table 2:** Main characteristics of the study population (n = 291)

	Mean ± SD
Age (years)	7.99 ± 1.55
Mother's educational level (years)	4.51 ± 1.43
Weight (kg)	27.63 ± 8.26
Height (cm)	126.28 ± 9.93
BMI‐for‐age (*Z*‐Score)	0.31 ± 1.31
Height‐for‐age (*Z*‐Score)	‐1.23 ± 1.22
Percentage body fat	29.12 ± 6.68
Stool leukocytes (CPF)	2.36 ± 1.52
C‐Reactive Protein (mg/L)	0.97 ± 1.60
Interleukin 6 (pg/mL)	3.12 ± 3.95
Interleukin 10 (pg/mL)	4.33 ± 6.82
Tumour necrosis factor‐α (pg/mL)	4.25 ± 2.87

SD, Standard Deviation; CPF, Cells per observation field.

After adjusting by sex age and mother's educational level, overweight/obese children were more likely to have higher concentrations of IL‐6 (aOR: 2.31 95% CI: 1.35‐3.93), TNF‐ α (aOR: 6.58 95% CI: 3.68‐11.76) and leptin (aOR: 119.71 95% CI: 32.331‐443.305) than normal weight children. In contrast, no association was found between overweight/obesity with CRP, IL‐10 and stool leukocytes.

Children with multiple infections (aOR: 10.69 95% CI: 3.62‐31.54) were more likely to have higher leptin concentrations as compared to parasite‐free children. IL‐6, lL‐10, TNF‐α or CRP were not associated with the presence of any of the studied parasites (Table 3).

**Table 3:** Logistic regression between parasite (specific) infection with systemic (acute and chronic) and local inflammation markers

	Soil‐transmitted helminths^34^		*Ascaris lumbricoides* 30		Protozoa (104)		*Entamoeba coli* 36		*Endolimax nana* 32		Multiple infections^36^	
	aOR	95% CI	aOR	95% CI	aOR	95% CI	aOR	95% CI	aOR	95% CI	aOR	95% CI
Systemic												
C‐Reactive Protein	0.76	(0.34‐1.69)	0.50	(0.34‐1.88)	1.48	(0.83‐2.61)	1.26	(0.57‐2.77)	1.28	(0.56‐2.91)	1.53	(0.73‐3.20)
Interleukin‐6	1.27	(0.58‐2.76)	1.11	(0.48‐2.55)	0.82	(0.47‐1.44)	0.53	(0.24‐1.18)	0.84	(0.37‐1.89)	0.99	(0.49‐2.01)
Interleukin‐10	1.01	(0.48‐2.15)	1.12	(0.50‐2.53)	1.10	(0.64‐1.90)	1.09	(0.51‐2.32)	0.94	(0.42‐2.07)	0.73	(0.35‐1.52)
Tumour necrosis factor‐α	0.75	(0.33‐1.71)	0.88	(0.36‐2.11)	1.03	(0.58‐1.83)	1.23	(0.55‐2.75)	0.73	(0.31‐1.72)	0.71	(0.33‐1.53)
Leptin	3.33	(1.06‐10.49)	2.43	(0.74‐7.97)	1.55	(0.70‐3.43)	1.67	(0.63‐4.42)	1.68	(0.60‐4.71)	10.68	(3.62‐31.54)^a^
Intestinal												
Stool leukocytes	6.16	(2.28‐16.68)^a^	5.91	(1.97‐17.70)^a^	3.21	(1.83‐5.60)^a^	8.46	(2.85‐25.14)^a^	1.10	(0.51‐2.36)	4.63	(2.169.92)^a^

Adjusted Odds ratio (aOR) (95% Confidence Interval), adjusted by sex, age, mother's educational level, stunting and % of body fat.

Cut off values: CRP: 0.366 mg/L; IL‐6:1.81 pg/mL; IL‐10: 3.16 pg/mL; TNF‐α: 3.50 pg/mL; stool leukocytes: 3 cells per field.

a Significative association using Bonferroni‐adjusted test of significance. Considering the number of comparisons made: 6 comparisons (0.05/6, α = 0.008).

Children infected with intestinal protozoa, STH, *A. lumbricoides, E. coli* and multi‐infections were more likely to have a higher level of stool leukocytes as compared with parasite‐free children (Table 3).

## 4 DISCUSSION

In the present study, specific intestinal parasites were associated with higher stool leukocytes and leptin concentrations, but not with the other systemic inflammation markers measured. These results provide new evidence concerning the relationship between intestinal parasitic infection with systemic and intestinal inflammation in a population with a high prevalence of overweight and obesity.

As reported in different studies, children with overweight and obesity (high body fat) were more likely to have higher concentration of the inflammatory markers IL‐6, TNF‐α and leptin.^29^, ^30^, ^31^ The mechanisms behind this association have been explored, discussed and explained previously elsewhere.^32^

In this study, CRP, IL‐6, IL‐10 and TNF‐α were not associated with any of the studied intestinal parasites, and these results are in line with other studies.^33^ For instance, Sanchez et al,^34^ found no association between STH and IL‐10 in a study in Honduran children. Also, de Gier et al^35^, did not find differences in acute phase protein or CRP in Cuban or Cambodian children between infected and non‐infected children with STH. Our results confirm the lack of association between systemic inflammatory markers and intestinal parasitic infection. The lack of association observed in these studies could be attributed to the strategies intestinal parasites have developed to remain unnoticed by the systemic immune response, such as immunological modulation and evasion.^36^

To our knowledge, this is the first study to evaluate the relationship between inflammation and intestinal parasites in a population where overweight and obesity are highly prevalent. This is relevant as excess body fat and weight promote systemic inflammation.^37^ In the studied population, children with overweight and obesity had higher concentrations of TNF‐ α, IL‐6 and leptin which may lead to an increased risk of other diseases such as hypertension and type II diabetes.^38^ However, the inflammation observed in the children who participated in the study is apparently not related to intestinal parasitic infection.

Children with multiple infections were more likely to have higher leptin concentrations when compared to parasite‐free children, even after adjusting for body fat content.^39^ Similarly, recent studies in animal models and in vitro have shown that intestinal parasitic infection may have an effect on blood leptin concentrations.^40^ In contrast, Karul, et al in a case‐control study evaluating 40 patients found no association between intestinal parasites and leptin concentration. However, Karul, et al did not adjust results for socio‐economic status, adiposity and age of the participants, which are well‐known factors affecting leptin concentrations and parasitic infection.^41^ The findings of the present study might be related to role of leptin as a hormone in the gut.^42^ Leptin has shown to prevent epithelial apoptosis and promoting tissue repair, which are required for mucosal defence against pathogens.^43^ Due to the study design, the complexity and the multiple roles of leptin in human metabolism, it is not possible to unravel the mechanisms or to determine the causality of the association. To fully understand the influence of intestinal parasites on leptin concentrations, multiple leptin measurements should be taken throughout the day, as well as before and after antiparasitic treatment, while taking in consideration sex, age and body fat‐related differences.^44^, ^45^, ^46^, ^47^

All the studied intestinal parasites were associated with higher stool leukocytes. Intestinal parasites are invasive, they require living space and in many cases, they physically harm the intestine. For instance, *A. lumbricoides* penetrates through the gut and migrates to the bloodstream causing tissue injury that might trigger an immune response in the gut.^48^ In contrast, the association of *E. coli* with faecal leucocytes is unexpected, as *E. coli* is considered a “non‐pathogenic” protozoa. Yet, children infected with this parasite were more likely to have a higher number of stool leukocytes than other pathogenic parasites such as *A. lumbricoides* or children with multiple infections. Thus, even though *E. coli* is non‐pathogenic, infection with this parasite may have implications related to immunological and inflammatory pathways that may have a long‐term effect on human health, particularly in obese/overweight individuals. Similarly to our results, stool leukocytes have been associated with pathogens, such as *Salmonella* and *Shigella*.^49^ The literature addressing the effect of STH and intestinal protozoa parasites on stool leukocytes is scarce. However, one study reported no association between hookworm infection and intestinal inflammation measured as faecal calprotectin concentrations.^35^ The results indicate that intestinal parasites might have the ability to evade systemic inflammatory reactions, but they fail to do so in the gut.

The present study has strengths and limitations that are worth mentioning. The cross‐sectional design of the study does not allow to distinguish between causal and non‐causal relationships between local and systemic inflammation markers with intestinal parasitic infection. We performed multiple comparisons that increase the probably of type 1 errors; still, the magnitude of the associations and the consistency of the results together with the Bonferroni test for multiple comparisons suggest this is not the case. The results of the parasitological examination were based on 1 stool sample per child; thus, the number of infected children may be underestimated. However, a single Kato‐katz has been widely used in epidemiological studies such as this one.^34^, ^50^ The count of stool leukocytes measures the number of cells which may lead to non‐systematic misclassification and does not differentiate between the different immune cell subtypes. Further research that includes techniques that provide information on the linage of the cells could give more insight into the specific inflammatory reaction associated with each parasite.

## 5 CONCLUSION

According to our results, intestinal parasitic infection was not associated with IL‐6, CRP or TNF‐α, markers related to obesity and chronic disease, but was associated with intestinal inflammation. In addition, STH infection and having multiple infections were associated with higher leptin concentrations. Further research is needed to evaluate the effect of different intestinal parasites in inflammatory pathways and chronic disease overtime.

## CONFLICT OF INTEREST

The authors report no conflict of interest.

## AUTHOR CONTRIBUTIONS

JLR, OPG, MCP and GAZ conceived and designed the study. MC, DR and GAZ carried out the fieldwork and laboratory assessments. OPG and GAZ analysed data and OPG, MCP, KP, and JLR gave important intellectual advice. All authors were involved in writing the paper and had final approval of the submitted and published versions.
